# Effects on upper masonry structures caused by double-line parallel shield cutting group pile construction

**DOI:** 10.1038/s41598-024-72902-1

**Published:** 2024-09-27

**Authors:** Xiang Zhu, Shi-ju Ma, Kai-rong Hong, Yong-gang Ding

**Affiliations:** 1 Department of Engineering, Huanghe Science and Technology College, Zhengzhou, 450000 China; 2 School of Civil Engineering, Henan University of Technology, Zhengzhou, 450001 Henan China; 3 School of Civil Engineering, Zhengzhou University of Technology, Zhengzhou, 450044 Henan China; 4 State Key Laboratory of Shield Machine and Boring Technology, Zhengzhou, 450001 Henan China; 5 China Railway Tunnel Group Co., Ltd., Guangzhou, 511458 Guangdong China

**Keywords:** Double-line parallel tunnel, Shield cutting group pile, Construction parameters, Masonry structure deformation, Measured analysis, Engineering, Civil engineering

## Abstract

The effects on the upper masonry structure and the construction parameters of shield cutting piles were studied during shield construction, focusing on a shield interval of Zhengzhou Metro Line 5. The study utilized the actual engineering case of left and right double-lane shields superimposed on cutting cement soil group pile composite foundations beneath masonry structures. Findings revealed that masonry structures within approximately 30 m (5.0 times the tunnel diameter) were impacted before and after shield cut pile construction, resulting in deflection and twisting deformations of houses along the central axes of the left and right tunnel lines. Implementation of “clay shock” grouting outside the shield shell, radial grouting through small conduits, shield tail synchronous grouting, and secondary reinforcement grouting effectively mitigated the disturbance caused by shield construction to the ground. When shield cut piles passed beneath masonry structures, pressure on the soil chamber, total thrust, and cutterhead speed were consistently controlled. Furthermore, the cutterhead torque was appropriately reduced, and slurry injection volume increased, contributing to better control of house settlement.

## Introduction

The shield method of tunnel construction has emerged as a predominant approach in urban subway tunnel projects, owing to its high degree of mechanization, safety, and minimal environmental impact. However, in densely populated old urban areas where buildings are closely situated, tunnel construction near existing building foundations can compromise their bearing capacity, posing challenges to the structural integrity of surrounding buildings. Extensive research has been conducted to investigate the effects of shield tunneling on buildings.

Various studies, including those by Sun et al.^1,2^, Chen et al.^3^, Wu et al.^4^, Dimmock et al.^5^, Camos et al.^6^, Breth et al.^7^, Frisehmann et al.^8^, and Forth et al.^9^, have delved into the responses of building foundations and superstructures during shield construction through field measurements on actual shield-underpassing building projects. Additionally, numerical analyses by Xie et al.^10^, Jiang et al.^11^, Giorgia et al.^12,13^, and Burd et al.^14^ have explored the intensity of damage to the ground surface and adjacent buildings by developing 2D or 3D finite element models for shield construction. The majority of the above studies have focused on the impacts of shield crossing construc-tion on adjacent buildings, but none of them have considered the special working condi-tions of direct cutting pile by shield.

Professor Yuan Dajun’s team^15–17^ carried out the first shield cutting reinforced concrete pile test at home and abroad, and carried out innovative research on the cutting pile mechanism and cutting pile tool configuration. Wang et al.^18^ and Peng et al.^19^ analyzed the force on the cutterhead of shield cutting pile and gave the calculation formula of load on the cutterhead of shield cutting pile. The above cutting pile objects are all reinforced concrete piles, and there are few studies on the construction of cutting cement soil pile. Therefore, it is of great practical engineering significance to evaluate building responses and shield construction parameter settings when left and right line shield superimposed cutting cement soil pile group underpassing.

Reinforced concrete piles are characterized by higher strength and toughness, whereas cement soil piles are relatively softer. Therefore, during the cutting process, reinforced concrete piles often require greater cutting force, while cement soil piles are more prone to deformation. This mechanical behavior difference is crucial for optimizing the configuration of shield tunneling tools and construction parameters. This study combined the experimentally measured data of a shield interval of Zhengzhou Metro Line 5 to monitor and analyze the deformation law of masonry structures and evaluated their safety. At the same time, the change laws of construction parameters when the shield cut under the composite foundation of cement soil group pile in Zhengzhou powder ground layer was explored, which included cutterhead torque, total thrust, excavation speed, cutterhead speed and pressure on soil chamber to obtain suggested values of construction parameters. The results of the research provided a construc-tion guide for similar projects in the future.

## Project overview

### Overview of tunnels and masonry structures

**Fig. 1:**
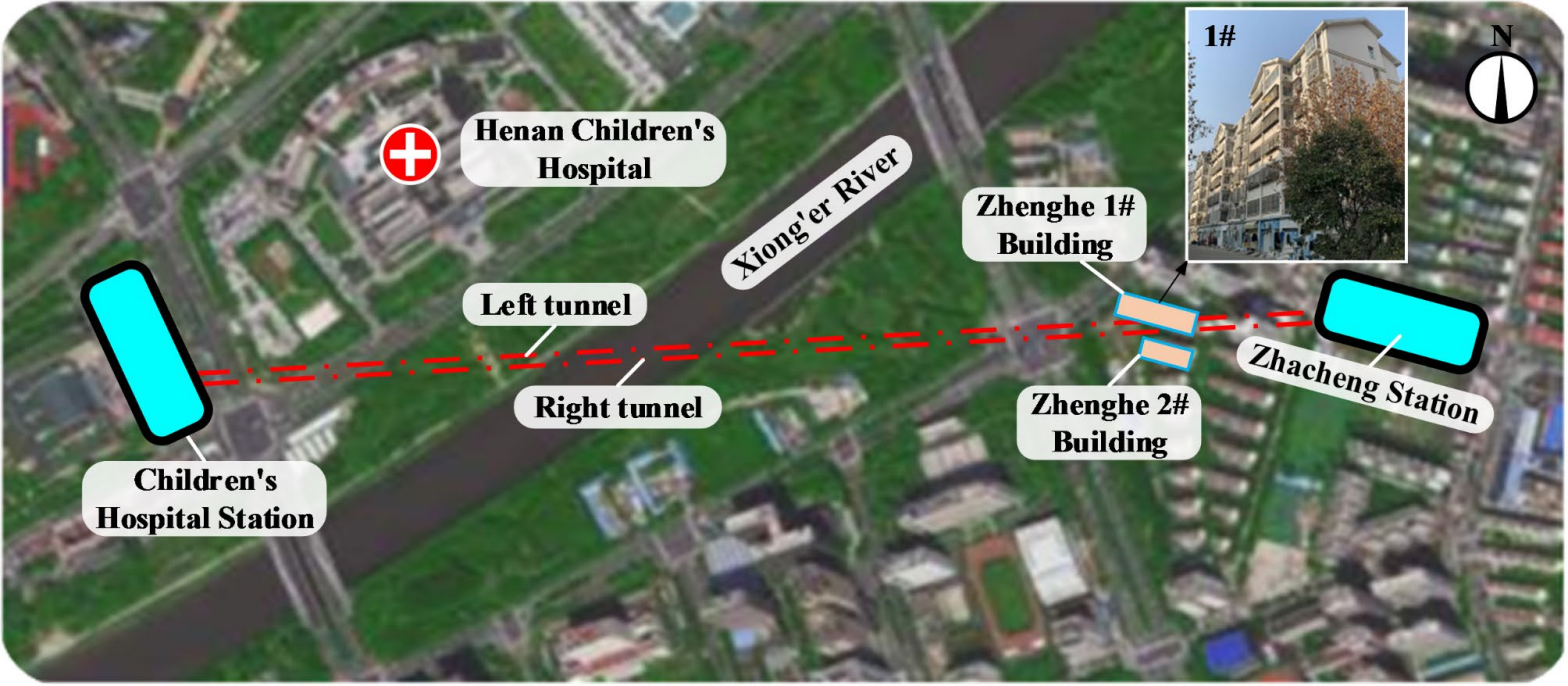
The location map of double line shield cutting group pile underpass masonry structure plan.

**Fig. 2:**
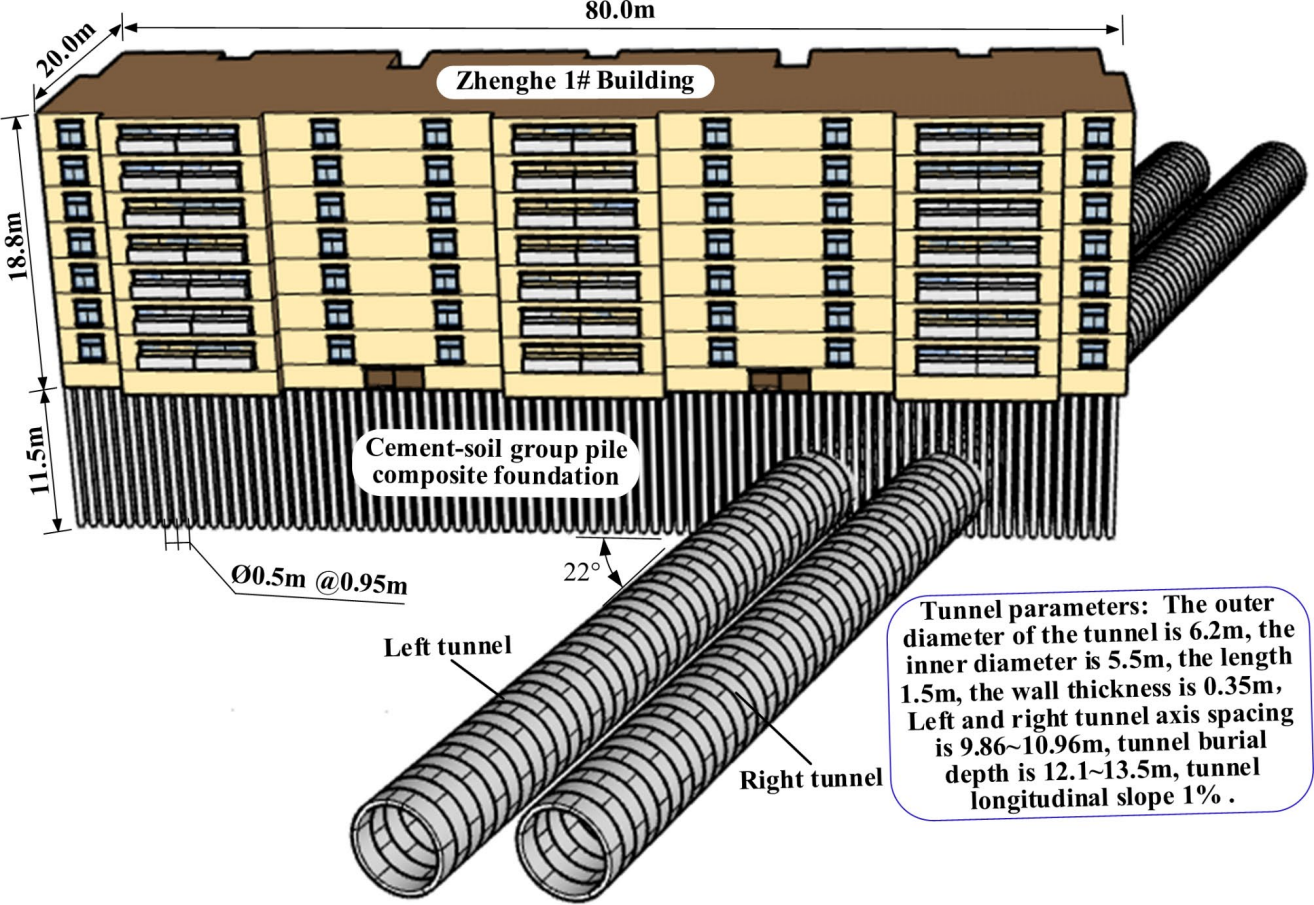
The relative positions of the masonry structure and the tunnel.

In a certain interval of Zhengzhou Metro Line 5, the left and right double-line shield superimposed cutting cement soil group pile composite foundations under the masonry structures of the project site (Fig. 1). The length of the shield cutting piles was about 2.6 ~ 3.7 m and the number of stirred piles invaded by shield in the left and right lines were about 224 and 114, respectively. The cut pile penetration mileage of left line was DK13 + 662.558 ~ DK13 + 711.322 (685 ~ 715 rings with length of about 48.764 m) and that of right line was DK13 + 694.410 ~ DK13 + 714.582 (705 ~ 719 rings with length of about 20.172 m). The tunnel crossed the building at an angle of 22° to the plane of the 7-story masonry structure which was completed in 2007. The foundation of the structure was of strip form and was treated by cement pile composite foundation and its material was C30 concrete. Also, bedding layer was C10 plain concrete of 0.1 m thickness with relative pile top elevation of − 4.3 m. Figure 2 illustrates an overview of the tunnel and masonry structure as well as their relative positions.

### Overview of engineering geology

**Fig. 3:**
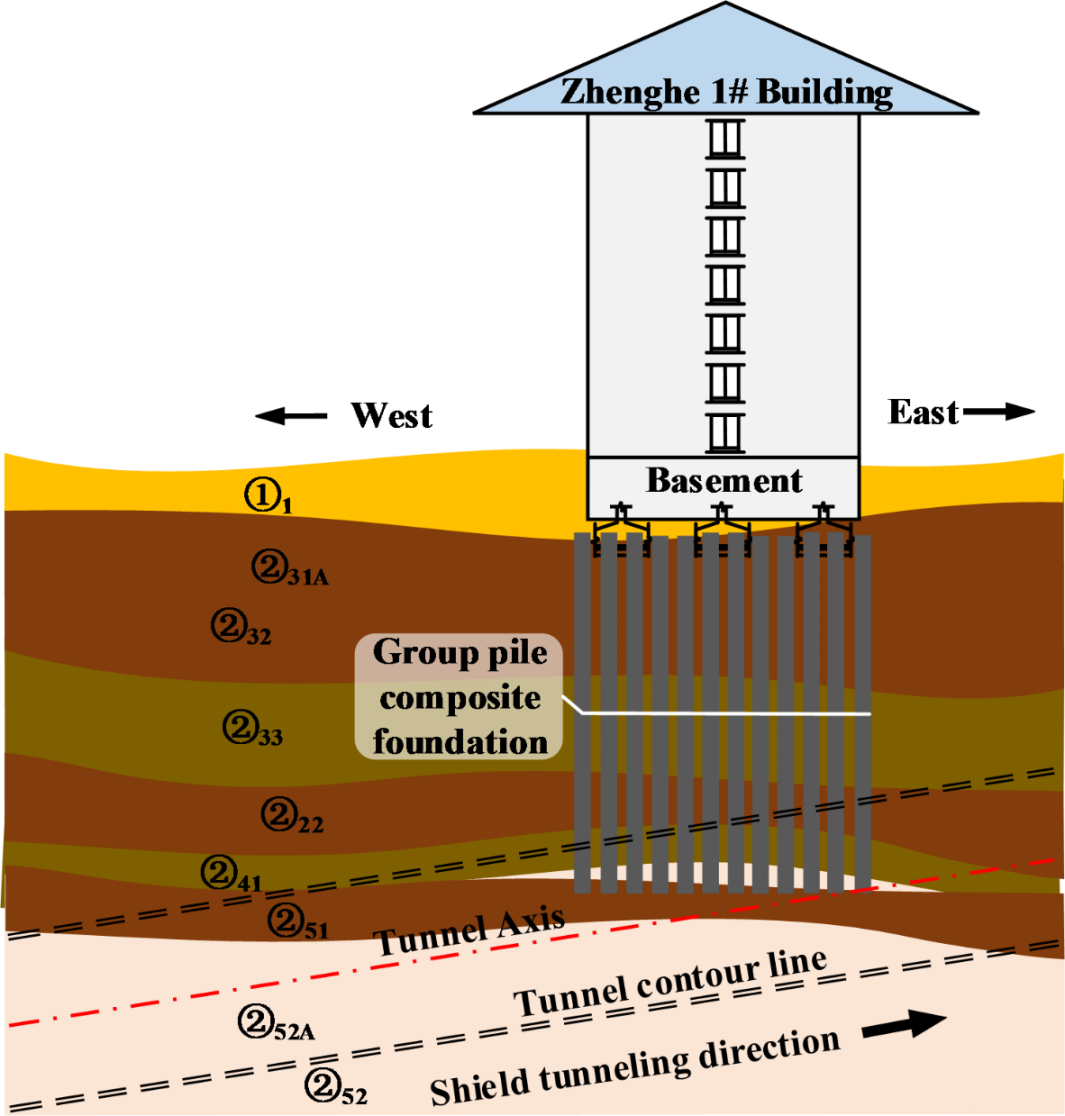
Engineering geological conditions.

Shield interval had a flat and open topography, belonged to Yellow River flood plain, and geomorphologically, it was a tertiary geomorphic terrace. The stratum within 30 m depth of construction site was mainly Quaternary Holocene (Q4) stratum; the main stratum from 0 to 20 m was composed of clayey silt and silty clay interspersed with chalk and fine sand and that from 20 to 30 m was consisted of medium-dense to dense fine sand. Figure 3 illustrates the typical geological section of shield crossing section at mileage DK13 + 680.226 and Table 1 summarizes the physical and mechanical parameters of each stratum.

**Table 1:** Physical and mechanical parameters of soil in each stratum.

Layer no.	Soil name	H (m)	ΔH (m)	W (%)	e	γ (kN/m^3^)	E_s_ (MPa)	µ	c (kPa)	Φ (°)	f_ak_ (kPa)
①_1_	Fill soil	0.0 ~ -2.7	2.7	18.0	0.95	20	4.5	0.33	20	20	110
②_31 A_	Clayey silt	-2.7 ~ -5.3	2.6	19.8	0.707	18.9	9.2	0.29	16.7	20.7	140
②_32_	Clayey silt	-5.3 ~ -8.2	2.9	20.8	0.676	19.4	8.5	0.30	16.9	20.6	130
②_33_	Silty clay	-8.2 ~ -9.2	1.0	25.1	0.73	19.6	5.7	0.36	25.5	14.3	120
②_22_	Clayey silt	-9.2 ~ -10.7	1.5	23.2	0.668	19.9	7.5	0.32	16.0	19.2	130
②_41_	Silty clay	-10.7 ~ -13.7	3.0	27.8	0.85	19.4	6.1	0.38	24.9	14.4	120
②_51_	Clayey silt	-13.7 ~ -16.2	2.5	21.1	0.625	20.2	8.7	0.33	16.2	19.3	140
②_52 A_	Fine sand	-16.2 ~ -22.1	5.9	2.1	0.7	19.4	20.0	0.27	1.0	30.0	200
②_52_	Fine sand	-22.1 ~ -27.9	5.8	2.0	0.7	19.5	22.0	0.27	1.0	32.0	220
③_23_	Silty clay	-27.9 ~ -33.9	6.0	19.5	0.594	20.3	9.8	0.30	29.7	15.3	230

*H* is depth; Δ*H* is the thickness of each layer; *w* is water content; *e* is void ratio; *γ* is saturated unit weight; *E*_*s*_ and *µ* are oedometric compression modulus and Poisson’s ratio, respectively; *c* and *φ* are the effective cohesion force and friction angle of drained type, respectively; and *f*_ak_ is characteristic value of bearing capacity.

## Masonry structure settlement control technology and monitoring scheme

### Masonry structure settlement control technology

**Fig. 4:**
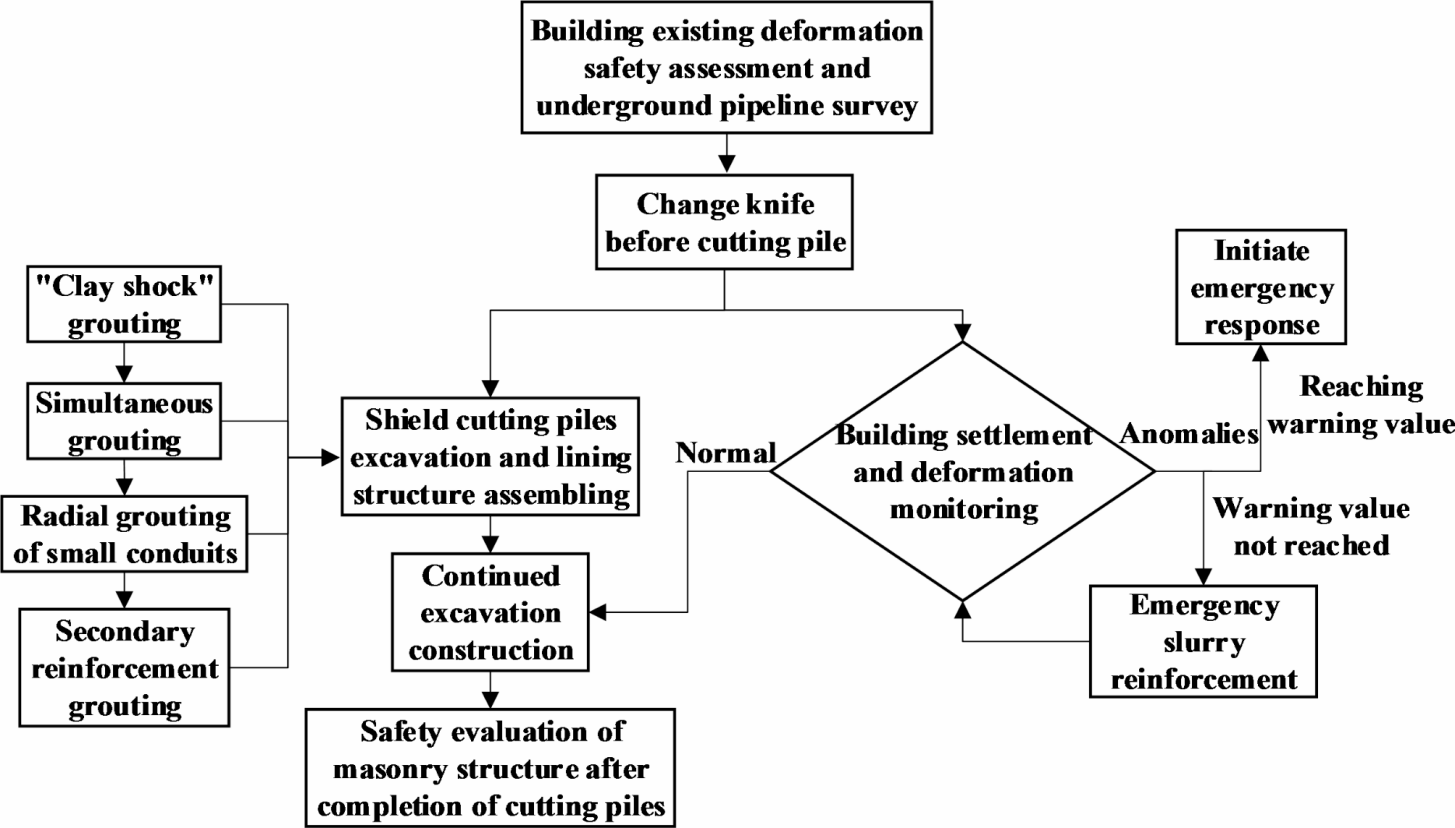
Flow chart of safe construction management measures for shield cutting piles.

**Fig. 5:**
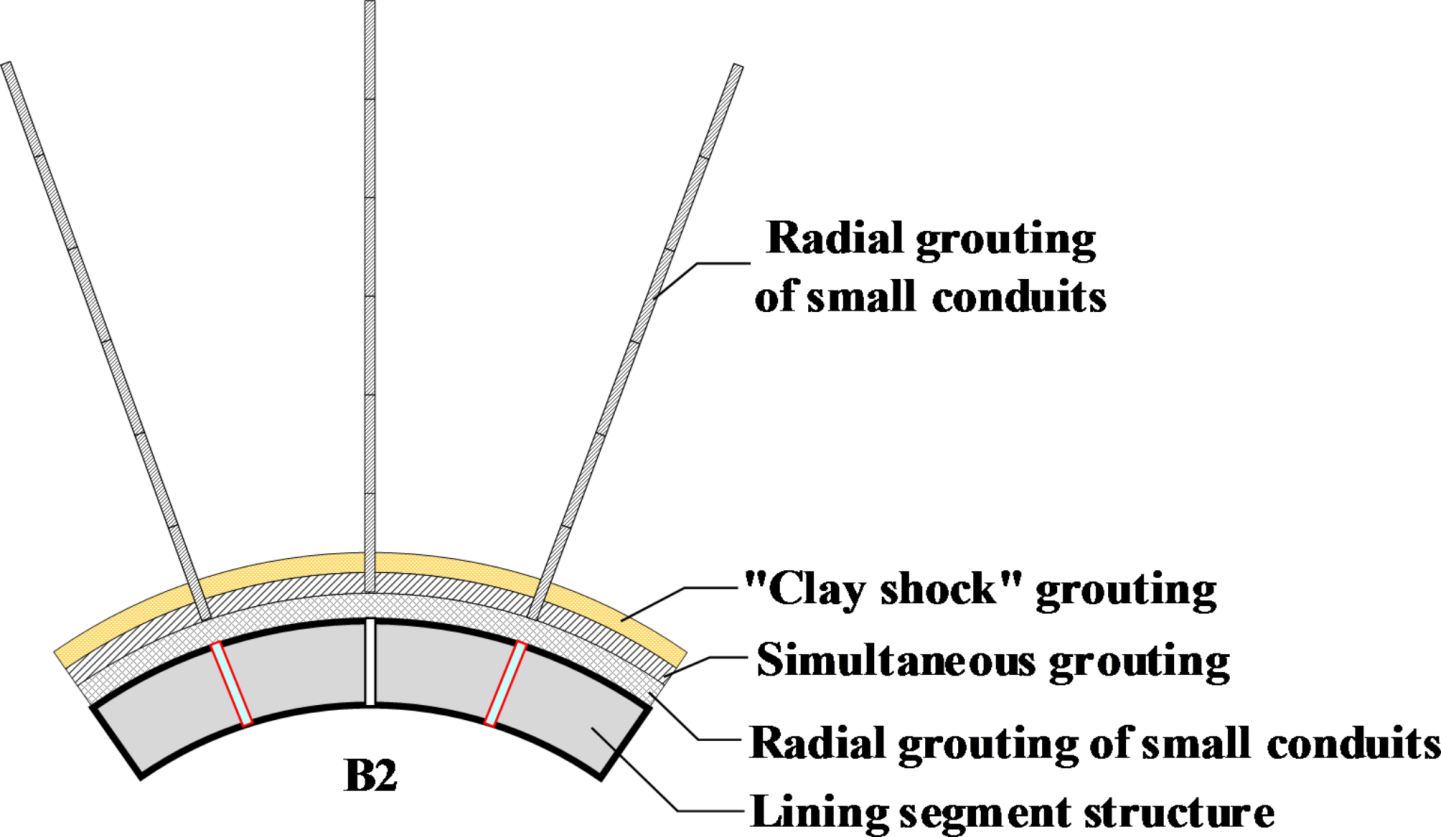
Diagram illustrating settlement control measures for B2 lining segment structure.

To minimize the impact of shield construction on the bearing capacities of composite foundations and upper masonry structures, four approaches were selected: “clay shock” grouting outside the shield shell, small conduit radial grouting, shield tail synchronous grouting, and secondary reinforcement grouting. These methods ensure that shield pile cutting construction can proceed smoothly without affecting the normal functions of the structures. Figure 4 illustrates the safety construction management measures for shield pile cutting, and the specific steps are as follows:

1. *Safety assessment of existing housing and underground pipeline deformations* Before commencing shield pile cutting near a building, a third-party unit with housing monitoring qualifications must perform a safety assessment of the affected house and provide a housing safety evaluation report. Additionally, the distribution of underground pipelines near the crossing section must be surveyed.
2. *Stopping and changing the cutter before pile cutting* The shield cutting pile excavation method primarily involves cutting, supplemented by rolling. It requires opening the chamber under normal pressure to change the cutter and increase the number of hob measures, directly using the hob to cut through the cement–soil mixed composite foundation piles.
3. *Initiating shield structure pile cutting and monitoring settlement and deformation* After changing the cutter, the shield structure begins pile cutting while simultaneously conducting real-time dynamic monitoring of building settlement and deformation. Ground monitoring information is promptly fed back to the central and shield tunneling control rooms.
4. *Commencing shield structure excavation* Four grouting reinforcement control measures—“clay shock” grouting, radial grouting of small conduits, synchronous grouting, and secondary reinforcement grouting—are implemented to mitigate composite foundation settlement. Figure 5 demonstrates the settlement control effect of the B2 lining pipe sheet structure.
5. *Continued tunneling construction* If house settlement and deformation remain within normal limits, the shield machine continues tunneling construction until pile cutting is completed.
6. *Handling abnormal settlement and deformation* If abnormal settlement and deformation occur, the situation is classified into two types: (a) If the warning value is not reached, emergency grouting (double slurry injection through additional grouting holes into the tunnel segment structure) is used for direct reinforcement. (b) If the warning value is reached, an emergency response program is immediately initiated, with the project manager acting as team leader and production leader. Emergency measures are carried out in an orderly manner, with support from the construction team and logistics staff. Once technical measures such as grouting normalize house settlement and deformation, the shield machine can continue excavation.
7. *Post-pile cutting safety evaluation* After pile cutting is completed, a safety evaluation of the masonry structure is conducted.

### Settlement monitoring scheme

**Fig. 6:**
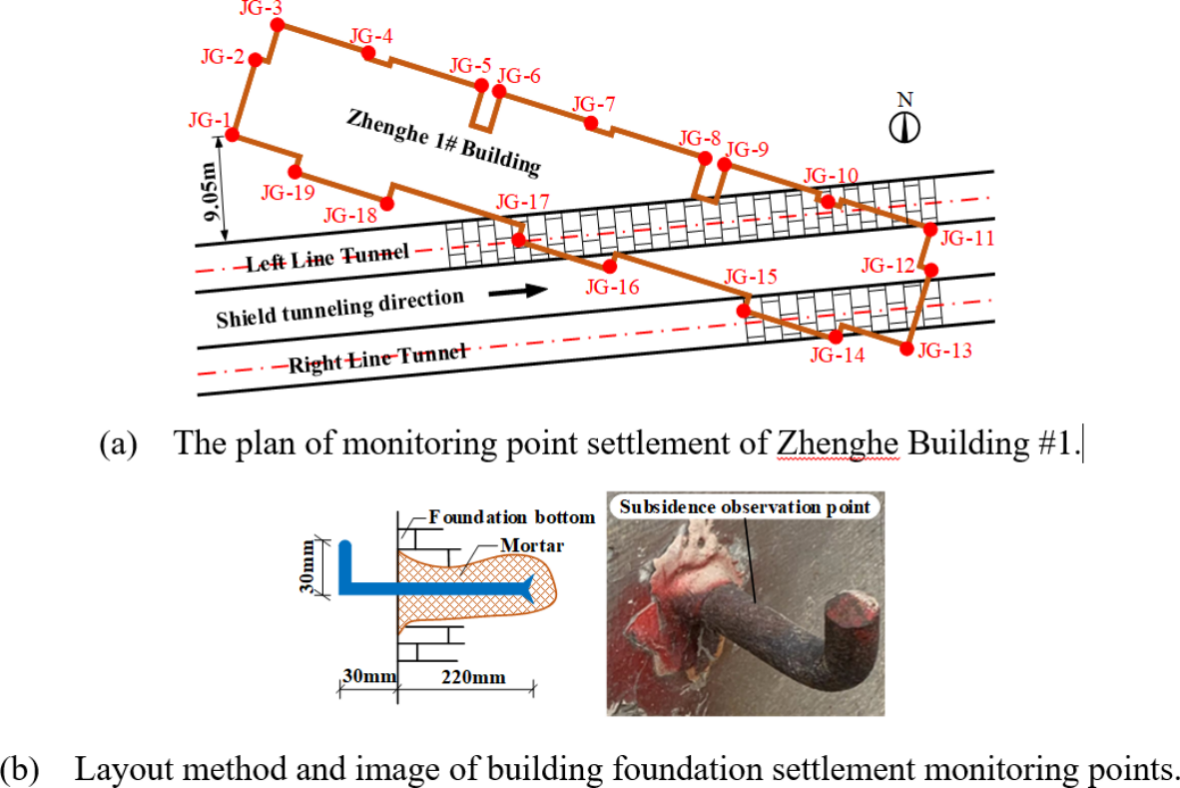
The layout and setting method of settlement measurement points.

The foundation of Zhenghe Building #1 was a of cement soil mixing pile composite foundation and the total number of mixing piles intruded by shield in the left and right lines was 338. Construction was carried out by adopting the scheme of shield machine directly cutting through piles, which was could decrease composite foundation bearing capacity and cause excessive deformation of masonry structure. The safety risk of construction was great with risk level being I risk source. In order to dynamically monitor masonry structure response during tunnel construction in real time, based on the monitoring requirements of “Technical Specification for Urban Rail Transit Engineering Monitoring”^20^ and “Specification for Urban Rail Transit Engineering Measurement”^21^, a total of 19 foundation settlement observation points labelled as JG-1 to JG-19 were laid at the bottom of building foundation along length and width directions. Figure 6 illustrates the details of the arrangement and setting method settlement measurement points.

## Responses of masonry structure during shield construction

### Settlement law of masonry structure

**Fig. 7:**
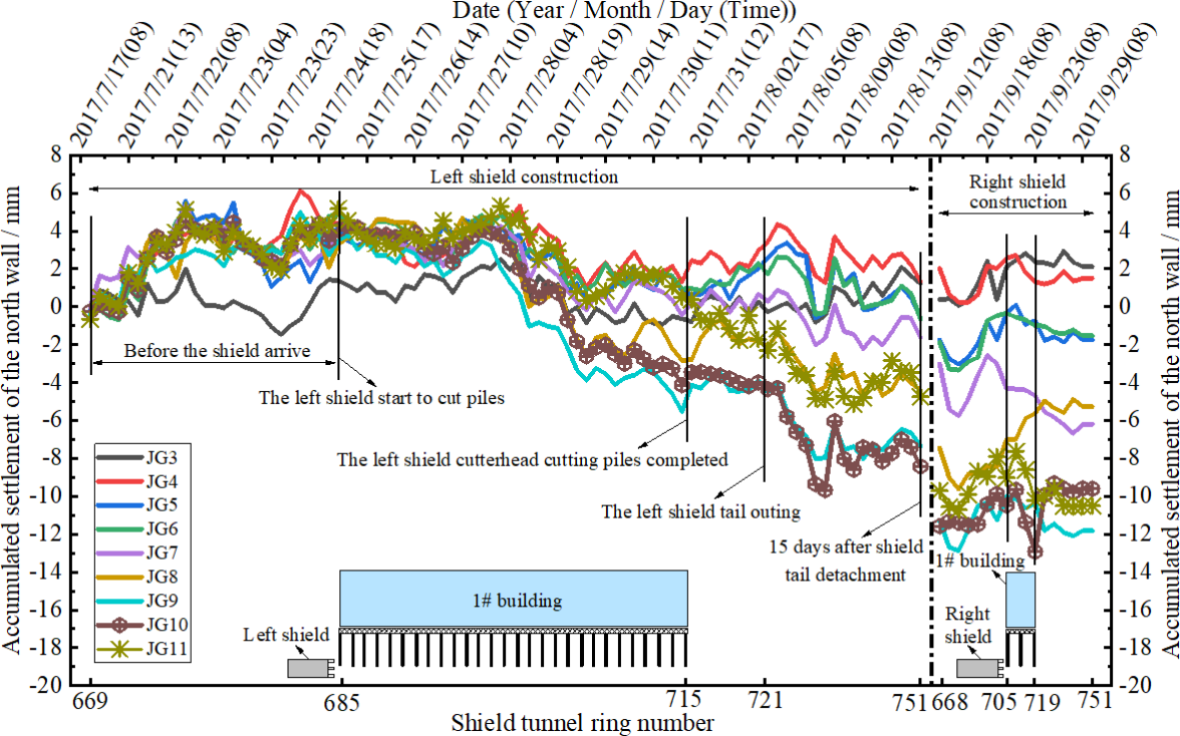
Settlement time history curves of the north wall measurement points of masonry structure.

**Fig. 8:**
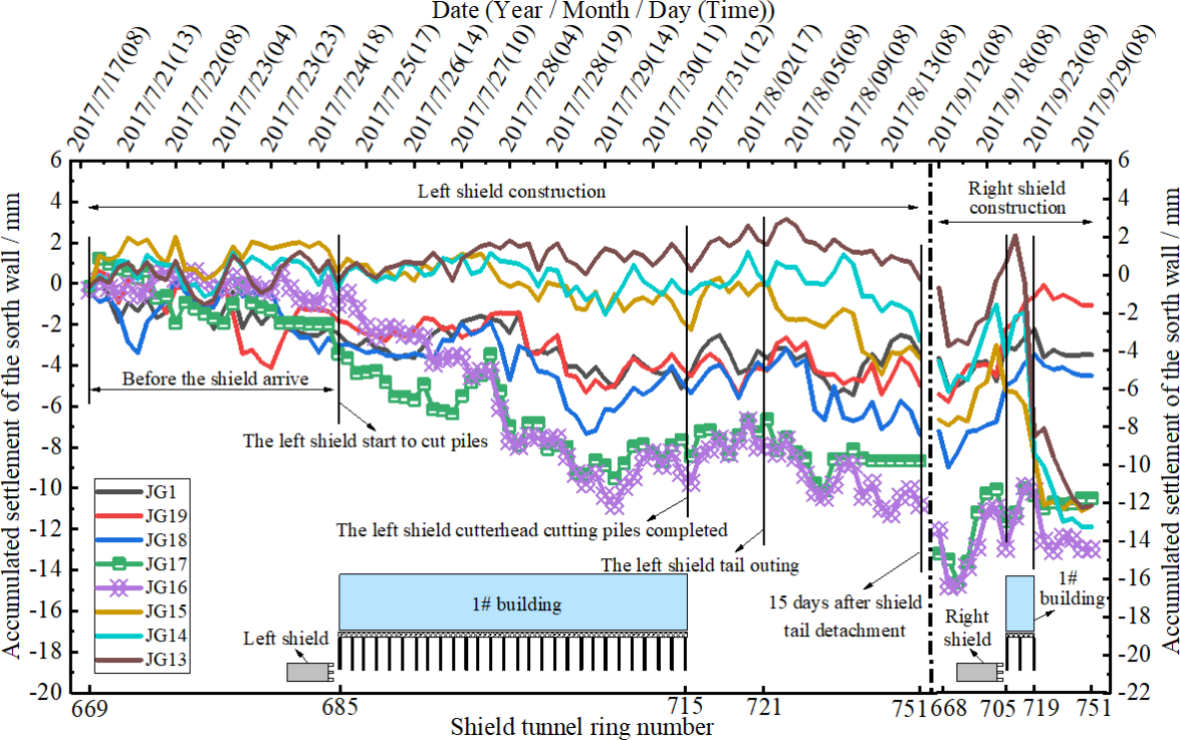
Settlement time history curves of the south wall measurement points of masonry structure.

Figures 7 and 8 depict the settlement-time curves of the north and south walls of the masonry structure during continuous shield tunneling left and right line pile cutting. From the figures, it can be observed that the settlement values at various points on the south wall are slightly larger than those on the north wall, but the variation range at the south wall points is smaller than at the north wall. The impact of left line shield tunneling pile cutting on the masonry structure is greater than that of the right line construction. This is mainly because the number of pile cuts by the left line shield tunnel is greater than that by the right line, resulting in additional stress on the composite foundation during left line construction being greater than on the right line. Therefore, the impact of left line shield tunnel construction on the bearing capacity of the composite foundation is greater than that of the right line. In addition, influenced by the number of pile cuts, the total thrust, cutter torque, and cutter speed of the left line shield tunnel are greater than those of the right line, which is conducive to continuous pile cutting. However, the impact of left line shield tunnel construction parameters on the rich water sand layer is significant, causing high groundwater flow and easily resulting in extensive stratum disturbance. Therefore, this study adopts “clay grouting effect” grouting to control the initial disturbance of shield tunneling construction on the stratum. Later settlement consolidation of the soil mass is controlled through synchronous grouting, secondary grouting, and small-diameter radial grouting measures.

Before the left line shield reached Building #1, all measurement points on the north wall of Building #1showed elevation with maximum value of + 5.59 mm occurring at measurement point JG-7. The measurement points of the south wall of Building #1slightly sank with maximum settlement value of − 2.21 mm occurring at measurement point JG-1 (southwest corner of Building #1). From the time the shield cut into the group pile composite foundation to the end of the time when the shield was released from the composite foundation, the whole cutting process of group pile composite foundation in the left line, the settlement measurement points of the building above the contour line of the left line, JG-17 and JG-16 measurement points of the south façade wall and JG-10 and JG-11 measurement points of the north façade wall were the most dangerous positions, in which the settlement change range of JG-17 and JG-16 measurement points of the south façade wall were − 1.98 ~− 7.51, + 0.34 ~− 7.51, and + 0.34 ~− 7.51 mm, respectively. 15 days after releasing the shield tail of the left line, the settlement values of JG-17 and JG-16 points on the south façade wall were measured to be − 8.66 and − 10.98 mm and those of JG-10 and JG-11 on the north façade wall were − 9.63 and − 7.76 mm, respectively. The significant rebound of 3 mm to 4 mm observed between ‘The left shield tail outing’ and ‘15 days after shield tail detachment’ is noteworthy. The main reasons causing this phenomenon are synchronous grouting at the shield tail and secondary grouting, which lead to slight uplift after the segment separates from the shield tail. Additionally, the higher soil pressure at the tunnel bottom compared to the tunnel top also contributes to this occurrence.

As the left line of the shield was far away from the building 60 rings, right line tunneling started to be bored and building settlement measurement points which were most affected by the right line pile cutting were still JG-17 and JG-16 of the building settlement above the left line tunnel contour line and JG-10 and JG-11 of the north façade wall. When the right line of shield cut pile penetrated Building #1, the maximum settlements of south façade wall at JG-17 and JG-16 were − 10.99 and − 13.06 mm and those for north façade wall at JG-10 and JG-11 were − 12.92 and − 10.56 mm.

Settlement during the pile-cutting underpass of the left shield line accounted for 45 ~ 60% of the maximum settlement and that during the pile-cutting underpass of the right shield line accounted for 20 ~ 35% of the maximum settlement. Settlement during the entire successively cut pile penetration of left and right lines accounted for 80-90% of the final settlement.

### Deformation law of masonry structure

**Fig. 9:**
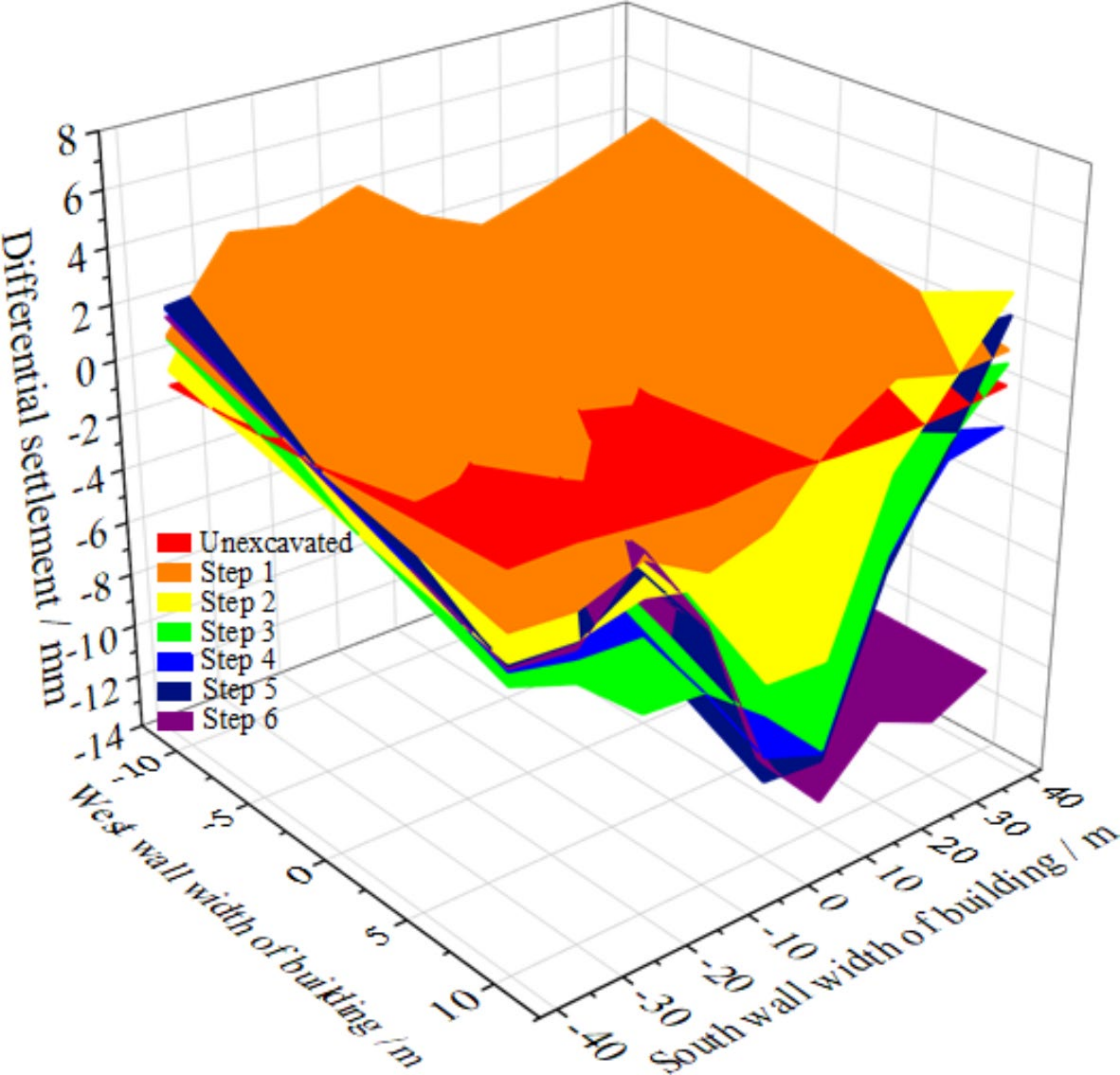
3D settlement deformation diagram of Building #1 under critical working conditions.

During the shield tunnel underpassing Building #1, there was a large time difference between the cutters reaching the two façade walls of the building, which caused inconsistent settlement generated on them and leading to permanent distortional deformation of the building. As illustrated in Fig. 9 and based on the time sequence of tunnel construction, six key working conditions were adopted including before and after the left line shield cut pile went down through Building #1 (Steps 1 and 2), after settlement stabilization due to left line construction (Step 3), before and after the right line shield cut pile went down through Building #1 (Steps 4 and 5) and after the settlement stabilization due to right line construction (Step 6, when the settlement surfaces of the left and right lines superimposed on the common response after cut pile construction).

It was seen from Fig. 9; Table 2 that the maximum differential settlement and distortion deformation of Building #1 occurred near the central axes of the left and right line tunnels and those of the south and north façade walls occurred at Step 5 with the values of 9.98 mm/m and 3.615E−2 rad/m, respectively. The settlement was finally stabilized after Step 6 and the maximum settlement, maximum differential settlement and maximum distortion values were recorded to be − 12.98 mm, 7.72 mm/m, and 3.34E−2 rad/m, respectively.

**Table 2:** Maximum differential settlement and maximum distortion values of building #1 under critical working conditions.

Monitoring parameter	Work conditions					
	Step 1	Step 2	Step 3	Step 4	Step 5	Step 6
Maximum differential settlement (mm/m)	7.43	8.18	8.42	8.37	9.98	7.72
Maximum distortion (rad/m)	1.65E^−2^	3.376E^−2^	3.368E^−2^	3.12E^−2^	3.615E^−2^	3.34E^−2^

### Safety evaluation of building # 1 based on existing deformation

Based on the damage level correction evaluation criteria proposed by Yang et al.^22^ taking into account building tilt and distortion, as summarized in Table 3 combined with the data given in Table 2, it was seen that after the construction of the left and right line shield successively superimposed cutting group pile composite foundation was completed, the maximum differential settlement of Building #1# was smaller than 10 mm/m and its maximum distortion value was lower than 4E−2 rad/m. It was revealed that the damage level of Building #1was medium and its risk level was 3. After a professional housing inspection agency tested and evaluated the building, it was proved that the building met normal use standard and could be used normally after local repair.

**Table 3:** Building damage grade evaluation standard.

Risk level	Maximum slope	Differential settlement (mm/m)	Building distortion (rad/m)	Risk description
1	< 1/500	< 2	< 4 × 10^−3^	Ignorable: shallow damage is unlikely
2	1/500 ~ 1/200	2 ~ 5	4 × 10^−3^ ~ 1 × 10^−2^	Minor: may be damaged in shallow parts, but has no effect on the structure
3	1/200 ~ 1/50	5 ~ 20	10^−2^ ~ 4 × 10^−2^	Moderate: Shallow damage, possible structural damage, possible related rigid pipe damage
4	> 1/50	> 20	> 4 × 10^−2^	Serious: building structure damage, rigid pipeline damage, possible other pipeline damages

## Statistical analysis of construction parameters

To investigate the change laws of main construction parameters before and after the pile-cutting construction of left and right shield lines, 20-ring distance before and after pile-cutting and construction parameters of shield machine during pile-cutting, mainly including pressure on soil chamber, total thrust, cutterhead torque, cutterhead speed and excavation speed, were statistically evaluated.

**Fig. 10:**
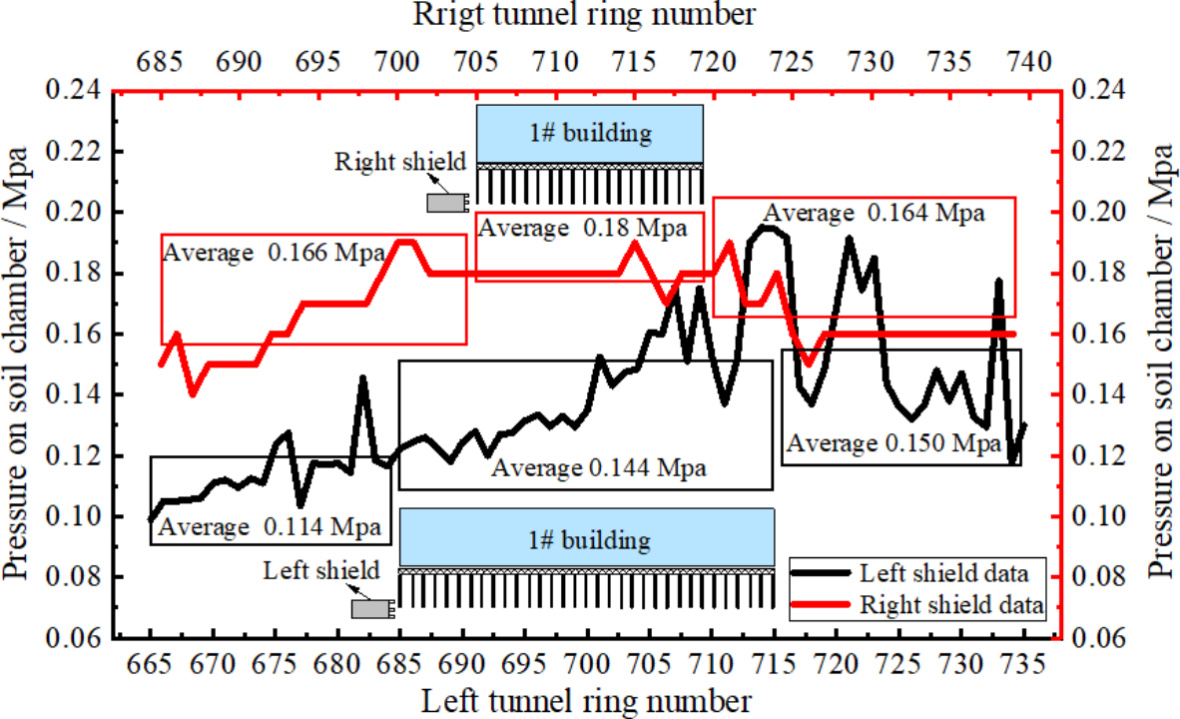
Pressure on soil chamber variation curves.

Figure 10 illustrates the pressure change curves of earth bin in left and right shield lines. The obtained results showed that the overall soil bin pressure was first increased and then decreased and its value in left shield line was lower than that in the right line. The reason for this was that pressure at shield palm surface in the left line was lower than that in the right line due to the effect of the number of cut piles and the additional stress of composite foundation which led to the same pattern of soil bin pressure. The average values of soil bin pressure during shield pile in left and right lines were obtained to be 0.144 and 0.18 MPa, respectively.

**Fig. 11:**
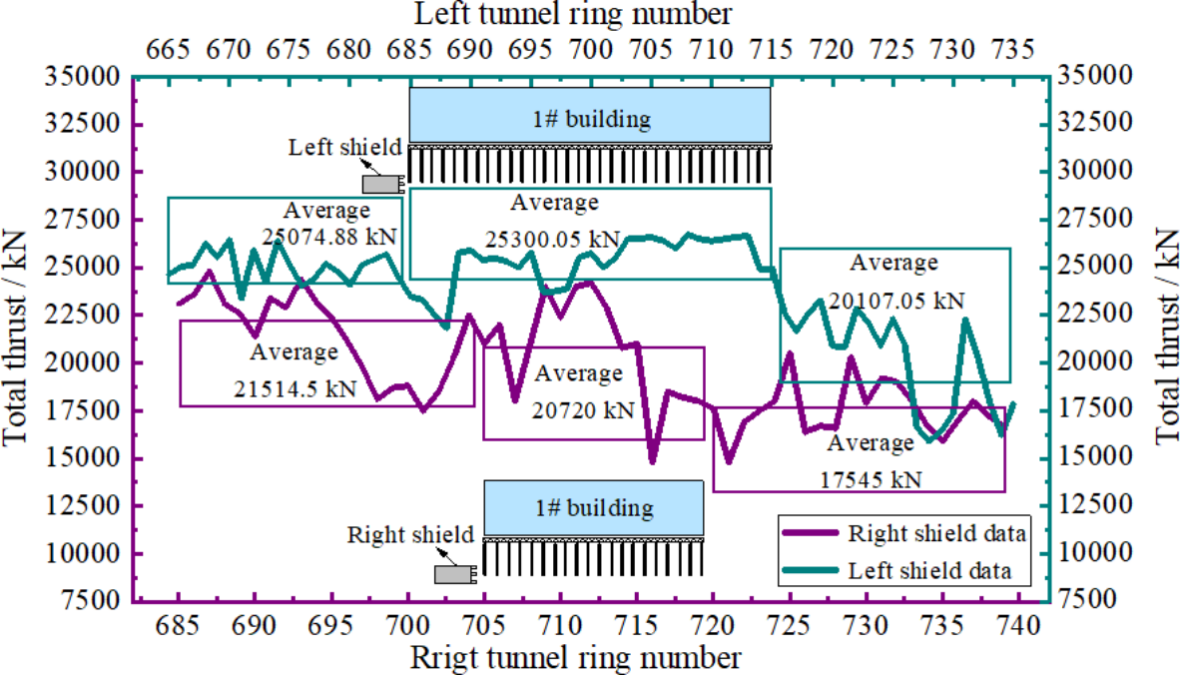
Total thrust variation curves.

Figure 11 illustrates the total thrust change curves of the left and right shield lines. Analysis showed that the overall total thrust presented a “W”-shaped pattern of decreasing, increasing and then decreasing, and the overall total thrust value of the shield in the left line was higher than that in the right line. The reason for this was that the left line had more pile cuttings and the additional stress on composite foundation was high. To limit pile deformation and its effect on composite foundation bearing capacity during shield cutting construction in the left line, shield total thrust in the left line was higher than that in the right line. The average values of shield pile cutting total thrusts during in the left and right lines were 25300.05 and 20720 kN, respectively.

**Fig. 12:**
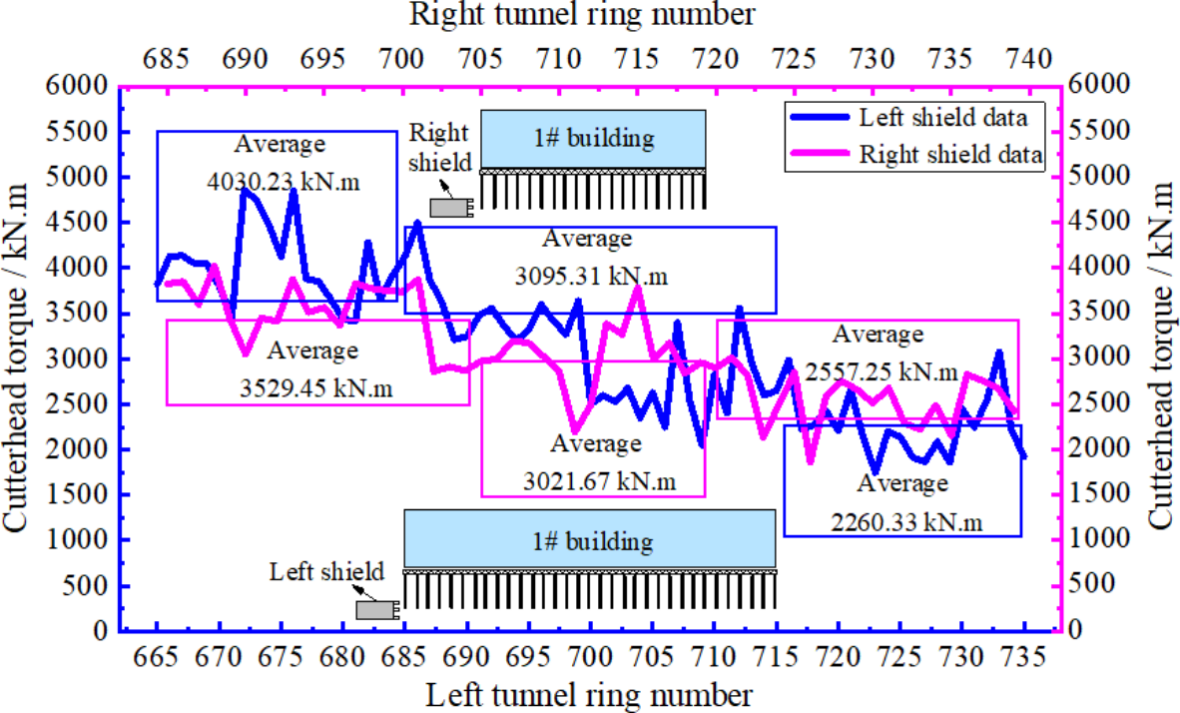
Cutterhead torque variation curves.

Figure 12 illustrates cutter torque change curves in the left and right shield lines. The analysis showed that cutter torque presented a gradual decrease and the overall cutter torque value in the left shield line was slightly higher than that in the right line. The reason for this was that the number of pile cut was higher in the left line and the appropriate increase of cutter torque could effectively improve pile cutting efficiency, but excessive cutter torque would also increase composite foundation disturbance. Therefore, cutter torque was gradually decreased. The average cutter torque values during pile cutting in left and right shield lines were 3021.67 and 3095.31 kN m, respectively.

**Fig. 13:**
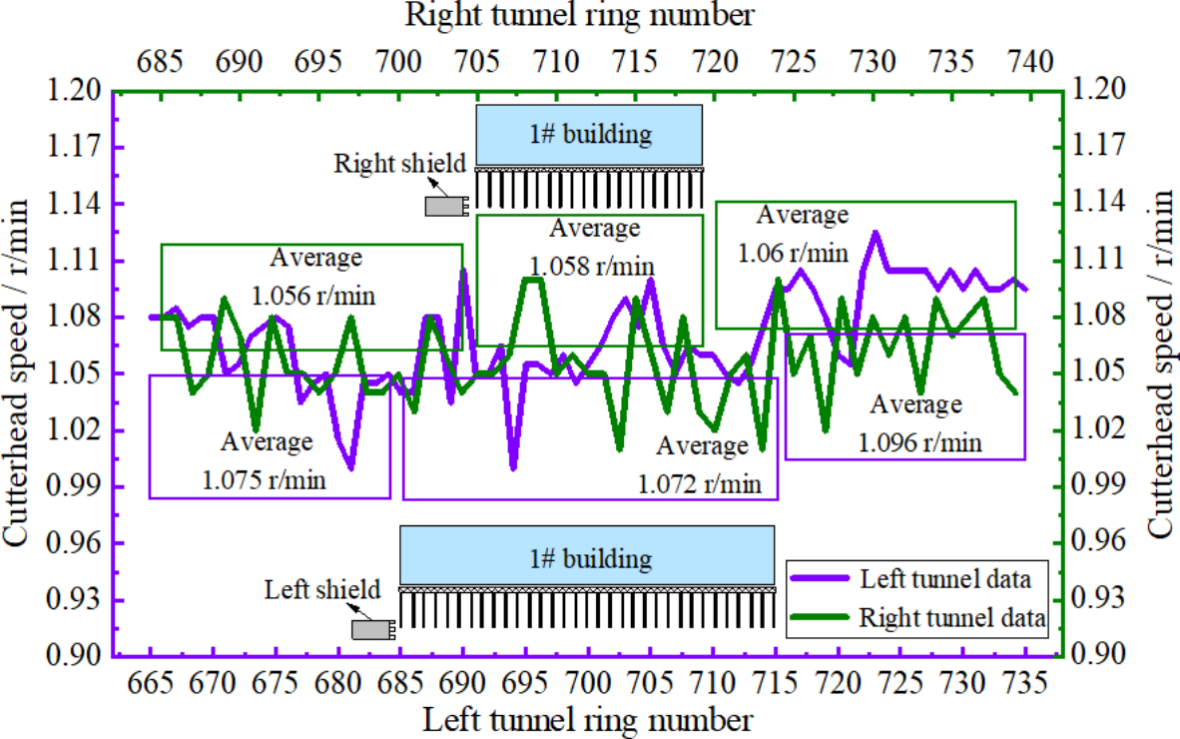
Cutterhead speed variation curves.

Figure 13 illustrates cutter speed change curves in the left and right shield lines. Analysis showed that the rotational speed of cutter plate presented a gentler trend and the overall rotational speed of shield cutter plate in the left line was slightly higher than that in the right line. The reason for this was that the number of pile cuts in the left line was higher and therefore, increasing the rotational speed of cutter could effectively improve pile cutting efficiency, but the disturbance to composite foundation would also increase at too high rotational speeds of the cutter. The average values of cutter rotational speed in shield cutting in the left and right lines were 1.058 and 1.072 r/min, respectively.

**Fig. 14:**
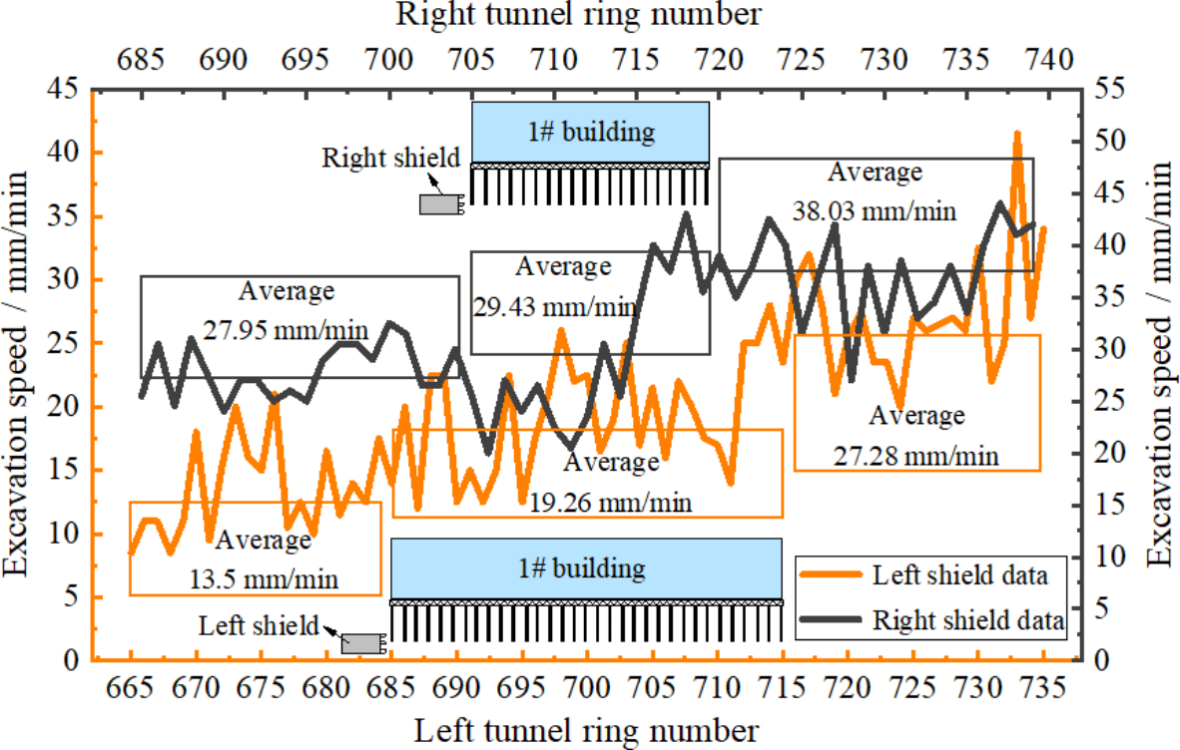
Excavation speed variation curves.

Figure 14 illustrates the change curves of the tunneling speeds of the left and right lines of the shield. Analysis showed that digging speed presented a gradually increasing trend and the overall value of shield digging speed in the left line was lower than that in the right line. The reasons for this could be that the number of cut piles in the left line was higher and excavation speed was too high to disturb composite foundation while it was too slow to cause large uneven settlement of composite foundation. Therefore, appropriate digging speed combined with four settlement control measures in this research ensured the safety of the above masonry structure. The average values of tunneling speed during shield pile cutting in the left and right lines were 19.26 m and 29.43 mm/min, respectively.

## Conclusions

Based on the data collected from the actual project of left and right double-lane shield superimposed cutting cement soil group pile composite foundation underpassing masonry structure, the settlement distribution characteristics and distortion deformation laws of the building during construction as well as shield construction parameters were analyzed in detail. The following main conclusions were drawn:

1. The effect range of masonry structure was about 30 m before and after shield cut pile (5.0 times tunnel diameter) and shield cut pile boring construction had the greatest influence on masonry structure. Evaluation of the uneven settlement and twisting deformation of the building revealed that the building deflected and twisted around tunnel central axis in the left and right lines, which needed to be controlled by certain construction tools and measures. The maximum settlement position appeared at the intersection of tunnel axis and building space; i.e., south wall measurement points JG-17 and JG-16, with values of − 8.66 and − 10.98 mm and north wall measurement points JG-10 and JG-11 with values of − 9.63 and − 7.76 mm, respectively.
2. In water-rich sand layer, the combined method of shield shell grouting, radial grouting with small guide pipes, simultaneous tail void grouting, and secondary reinforcement grouting can effectively mitigate the impact of shield tunneling on the strata, composite foundation, and superstructures above. Furthermore, prior to shield cutting through piles, appropriately reducing the total thrust, disc cutter torque, and disc cutter speed while increasing the earth pressure in the chamber can minimize uplift deformation of buildings. When the shield cuts through masonry structures, controlling chamber pressure, total thrust, and disc cutter speed, and appropriately reducing disc cutter torque while increasing grout volume, is more conducive to controlling building settlement. After shield cutting, significant adjustments to the shield construction parameters should be avoided.

## Data Availability

Some or all data, models, or code that support the findings of this study are available fromthe corresponding author upon reasonable request. These items include detailed output for all analyzed scenarios.
